# New Parasite Records for the Sunfish *Mola mola* in the Mediterranean Sea and Their Potential Use as Biological Tags for Long-Distance Host Migration

**DOI:** 10.3389/fvets.2020.579728

**Published:** 2020-10-19

**Authors:** Mario Santoro, Marialetizia Palomba, Simonetta Mattiucci, David Osca, Fabio Crocetta

**Affiliations:** ^1^Department of Integrative Marine Ecology, Stazione Zoologica Anton Dohrn, Naples, Italy; ^2^Department of Public Health and Infectious Diseases, Sapienza University of Rome, Rome, Italy

**Keywords:** *Anisakis simplex* (s.s.), Molidae fishes, Tyrrhenian Sea, *Gymnorhynchus isuri*, metazoan parasites, Trypanorhyncha

## Abstract

Studies describing the parasite fauna of sunfish species from the Mediterranean Sea are to date limited, despite information gained through parasitological examination may reveal unknown ecological and biological aspects of both hosts and parasites. Moreover, recent molecular studies on sunfish taxonomy revealed the presence of two species belonging to the genus *Mola* in the Mediterranean basin, namely *M. mola* and *M. alexandrini*. These two fish taxa have long been synonymized or confused among them, which implies that the majority of the studies carried out so far reported the parasites infecting both species under a single host species, generally referred to as *M. mola*. We hereby investigated the parasite fauna of a 43 cm long *M. mola* specimen from the Mediterranean Sea, whose identification was confirmed by molecular tool, and provided the first evidence of the occurrence of the nematode *Anisakis simplex* (s.s.) and of the cestode *Gymnorhynchus isuri* in *Mola* species anywhere. The use of helminth species as biological tags for the sunfish is also discussed.

## Introduction

The sunfish genus *Mola* Koelreuter, 1766 has been considered, until recently, as composed of two species, namely *Mola mola* (Linnaeus, 1758), with a cosmopolitan distribution, and *Mola ramsayi* (Giglioli, 1883), restricted to the southern hemisphere (1). In the past few years, the use of molecular approaches revealed the presence of three valid species within this genus: *Mola alexandrini* (Ranzani, 1839) (syn. *M. ramsayi*) and *M. mola*, with a wide and sympatric distribution in the world's oceans, including the Mediterranean Sea, and *Mola tecta* Nyegaard, Sawai, Gemmell, Gillum, Loneragan, Yamanoue and Stewart, 2017, mostly found from temperate waters of southern hemisphere (2–4).

*Mola* species are well-known to host a variegate parasite fauna, and in particular several papers focused in the past on the parasite fauna of “*M. mola*,” whose information was reviewed by de Figueiredo et al. (5). However, since the clarification of the worldwide taxonomy of the genus *Mola*, only Ahuir-Baraja et al. (6) investigated the endo-parasites of a *Mola* individual stranded on the Valencian coast (western Mediterranean Sea), identified by molecular tools as *M. alexandrini*. This likely implies that the majority of the studies carried out so far reported the parasites infecting the entire genus under the single species *M. mola* [see (5)].

Host specificity is often considered to be a result of various factors, including phylogenetic, physiological, and ecological aspects (7, 8). Since most marine parasites exhibit at least some degree of host-specificity or host-preference, it is plausible that a degree of host-specificity may exist for parasites infecting fishes of the genus *Mola*. Moreover, among the several methods applied to obtain biological and ecological data of fishes, the use of parasites as biological tags in marine environment has become a useful tool in producing data for the host stock identification and for studying their migration by using molecular tools to unequivocally identify both hosts and their parasites (9–12). In fact, parasites can be used as biological tags since their geographic ranges are definite, so that a host can only become infected when entering the parasite endemic range (9, 10).

We here investigated the whole parasite fauna of a *M. mola* specimen obtained from the Gulf of Naples (Tyrrhenian Sea) whose identification was confirmed through molecular analysis. We provide the first evidence in *Mola* species of the occurrence of larval forms of two helminth parasites typically known from the Atlantic Ocean, suggesting a potential long-distance migration of the examined host fish.

## Materials and Methods

### Sampling

A juvenile male *Mola* specimen (4,190 kg in weight; 43 cm in total length) was fished at ~50 m depth on January 3, 2020 off Ischia Porto (~40.753766, 13.947723, Ischia Island, central Tyrrhenian Sea, Mediterranean) by an amateur fisherman, using a fishing line with the Mediterranean mussel (*Mytilus galloprovincialis* Lamarck, 1819) as bait. Once the fish was landed, it was first shown to the public and then discarded, being considered as bycatch. As one of the authors (FC) was there at that moment, and the specimen was already dead, it was collected by him and soon after frozen at −20°C for molecular and parasitological analyses.

### DNA Extraction and Molecular Analysis for Host Identification

Total genomic DNA was extracted from a defrosted muscle sample, and a partial sequence of the cytochrome c oxidase subunit I (COX1) mitochondrial gene (mtDNA *cox1*) was amplified and sequenced following methods used in Osca et al. (13).

The sequence obtained was assembled using Sequencher v. 5.0.1 (GeneCodes Co.) and compared with reference sequences from the NCBI nucleotide (NT) database using BLASTn (14). Partial sequences of further *Mola* specimens were downloaded from GenBank and BOLD, together with those of *Masturus lanceolatus* (Liénard, 1840) to be used as outgroup (2). Nucleotide sequences were aligned with the Translator X server (15), using the MAFFT v7 (16) option with default settings. By subsequently deleting identical sequences, a total of 12 sequences of *Mola* specimens and of five of *M. lanceolatus* were used for the phylogenetic analyses (Supplementary Table 1).

Alignment format conversions were performed using the ALTER webserver (17). The best-fit model of substitution was determined using the Akaike information criterion (AIC) (18) implemented in PartitionFinder v.1.1.1 (19). Phylogenetic relationships were inferred performing maximum likelihood (ML) analyses in RAxML v.8.1.16 (20), using the rapid hill-climbing algorithm, and Bayesian inference (BI) analyses in MrBayes v.3.1.2 (21), running four simultaneous MCMC (Markov chain Monte Carlo) for 2 million generations, a sampling interval every 1,000 generations, and a burn-in of 25%. Two independent Bayesian inference runs were performed to increase the chance of adequate mixing of the Markov chains and of detecting failure to converge. Support for internal branches was evaluated by non-parametric bootstrapping (22) with 1,000 replicates (ML) and by posterior probabilities (BI). Finally, based on the results of the phylogenetic trees, diagnostic nucleotides were identified in our alignment as to further confirm the molecular identification of the specimen studied here.

### Parasitological Analysis

During the fish necropsy, skin, musculature, gills, mouth cavity, digestive tract, liver, heart, testes, visceral cavity, and mesenteries of the sunfish were examined for metazoan parasites. Organs and tissues were removed and placed individually in plastic Petri dishes (200 mm in diameter); the organs were then dissected and the surfaces were examined visually. After the larger helminths were removed using tweezers, organs and tissues were washed through a 100 μm mesh screen. The remaining washed material from each organ was examined under a dissecting microscope (Leica M165 C), and parasites were collected, counted, washed in physiological saline, and preserved in 70% ethanol or frozen at −20°C (23). For identification, crustacean parasites and nematodes were clarified respectively in 20% potassium hydroxide and Amman's lactophenol, and then returned to 70% ethanol; digeneans and cestodes were stained with Mayer's acid carmine and mounted in Canada balsam. Parasites were studied with a compound microscope (Leica DM1000) and identified using published identification keys (24–27).

### DNA Extraction and Molecular Analysis for Selected Larval Parasites

Total genomic DNA from a cestode and a nematode larva whose morphological identification at species level proved to be uncertain, was extracted using Quick-gDNA Miniprep Kit (ZYMO RESEARCH) following the standard manufacturer-recommended protocol. DNA was quantified by a NanoDrop®TC1-E20 spectrophotometer (BioTek Synergy HT).

A cestode larva morphologically considered as belonging to the family Gymnorhynchidae Dollfus, 1935 (Trypanorhyncha) was identified to species level by sequence analysis of the complete small subunit of the ribosomal RNA gene (ssrDNA) (28) and the partial large subunit ribosomal gene (lsrDNA) (29). Complete ssrDNA was amplified using the primers WormA (5′-GCGAATGGCTCATTAAATCAG-3′) and WormB (5′-CTTGTTACGACTTTTACTTCC-3′). Partial 1srDNA was amplified using the primers ZX-1 (5′-ACCCGCTGAATTTAAGCATAT-3′) and 1500R (5′- GCTATCCTGAGGGAAACTTCG-3′). Both PCR reactions were carried out following the procedure previously reported in Palm et al. (30). The identity of the specimens was checked using the Basic Local Alignment Search Tool (Blast, www.ncbi.nih.gov/BLAST/) and the sequences of ssrDNA and 1srDNA were aligned with the Gymnorhynchidae reference sequences from the NCBI nucleotide (NT) database, using Clustal X (31). The aligned data were concatenated using sequence Matrix v.1.7.8 (32). The phylogenetic analysis of the combined ssrDNA and 1srDNA sequences were carried out by BI, using MrBayes 3.1 (21). JModeltest (33) was used to determine the best-fit substitution model for both sequences dataset (ssrDNA and 1srDNA), as implemented with Akaike's Information Criterion (AIC). BI analysis was performed using the Bayesian posterior probability analysis using the MCMC algorithm, with four chains, 0.2 as the temperature of heated chains, 2,000,000 generations, with a subsampling frequency of 500 and a burn-in fraction of 0.25. Posterior probabilities were estimated and used to assess support for each branch. The phylogenetic trees were rooted using *Hepatoxylon trichiuri* (Holten, 1802) Bosc, 1811 as outgroup.

The *Anisakis* larva was identified at the species level by sequencing the mitochondrial cytochrome c oxidase subunit II (COX2) gene (mtDNA *cox2*). PCR amplification was performed using the primers 211F (5′- TTTTCTAGTTATATAGATTGRTTTYAT-3′) and 210R (5′-CACCAACTCTTAAAATTATC-3′). PCR was carried out according to the procedures provided by Mattiucci et al. (34). The sequences obtained at the mtDNA *cox2* for the larval nematodes were compared with those already obtained for the same gene and deposited in GenBank.

## Results

### Molecular and Phylogenetic Analyses for Host Identification

A 606 bp partial sequence of the mtDNA *cox1* gene was obtained for the fish specimen. The ML (*-lnL* = 1322.1) and BI (*-lnL* = 1356.15 for run 1; *-lnL* = 1353.81 for run 2) analyses arrived at similar tree topologies. Two major clades were defined with high support for both analyses (ML and BI), in which *M. tecta* and *M. alexandrini* were placed as sister groups. The other clade, with high support for ML and maximal support for BI, included sequences of *M. mola*. The latter clade also included the sequence of the specimen studied here (Supplementary Figure 1). The analysis of the positions of diagnostic nucleotides in our alignment revealed that *M. tecta* is diagnosed by 45 (G), 474 (A), 558 (G), and 576 (T), *M. alexandrini* is diagnosed by 168 (G), 240 (A), 291 (T), 516 (A), 597 (A), and 603 (G), and *M. mola* is diagnosed by 15 (A), 150 (T), 172 (G), 186 (C), 219 (C), 225 (A), 339 (T), 387 (C), 426 (G), 429 (C), 450 (G), 456 (A), 465 (G), 498 (A), 540 (G), 543 (T), 579 (T), and 606 (T). No differences with respect to present results were obtained when analyzing the unpublished *cox1* sequence of *M. mola* obtained here. The sequence was deposited in GenBank under the accession number MT913440.

### Parasitological Analysis

**Figure 1:**
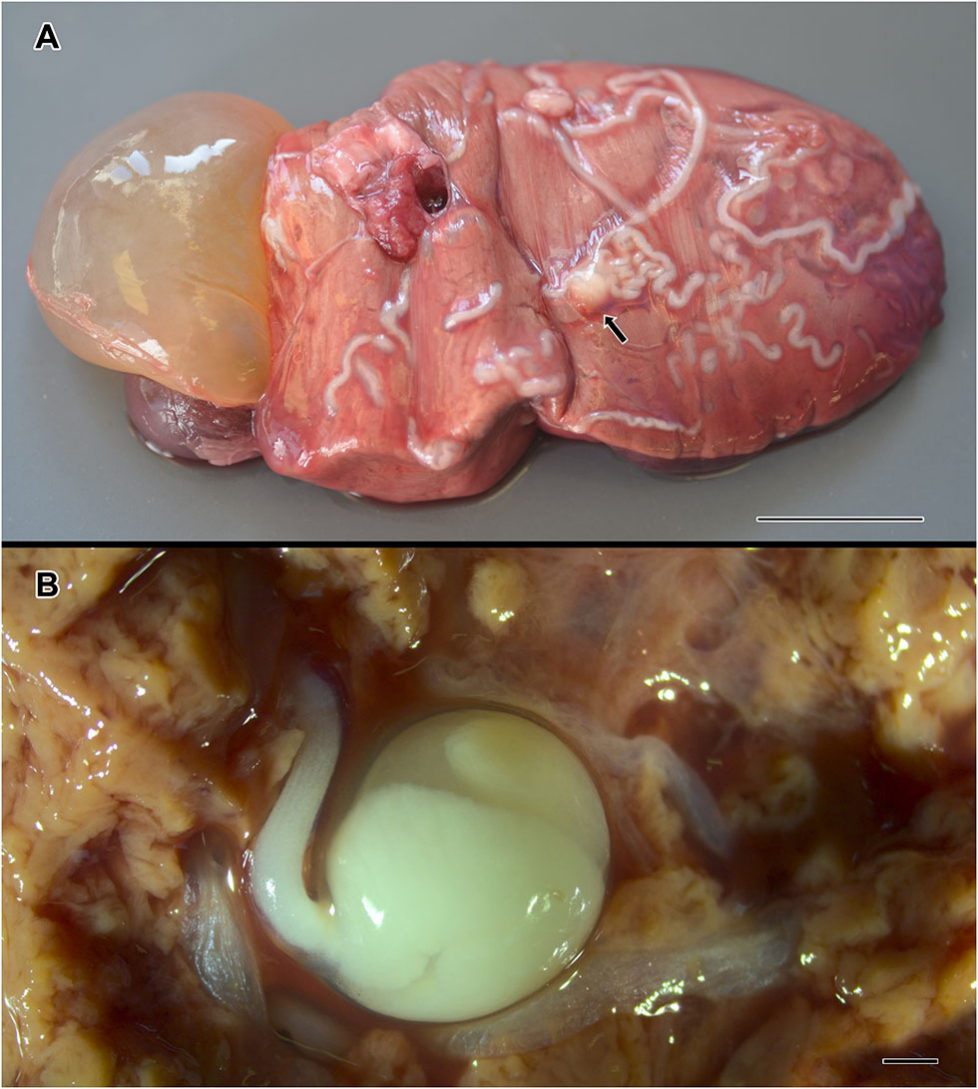
*Gymnorhynchus isuri* infection in the liver of the sunfish (*M. mola*). Several white larvae are embedded in the liver **(A)**; the arrow in **(A)** indicates the blastocyst of a *G. isuri* larva enlarged in **(B)** after disruption of the hepatic tissue. Bar scale: **(A)**: 2 cm; **(B)**: 100 μm.

A total of 46 metazoan parasites were found, belonging to 10 different species. These included eight helminth taxa (one monogenean, three digeneans, three cestodes, and one nematode) and two copepods (Table 1). All parasites were found at adult stage, except for the larvae stages of the cestodes *H. trichiuri* and *Gymnorhynchus isuri* Robinson, 1959 (Figure 1) and the nematode *Anisakis simplex* (s.s.) (Rudolphi, 1809) (see below for the molecular identification of the selected larval parasites). Among the ectoparasites, the monogenean *Capsala martinierei* Bosc, 1811 and the copepods *Cecrops latreillii* Leach, 1816 and *Lepeophtheirus nordmanni* (Milne Edwards, 1836) were among those found on gills and skin.

**Table 1:** Metazoan parasites found in a sunfish *Mola mola* from off Ischia Island (Tyrrhenian Sea, Mediterranean Sea), with number of individuals (N), developmental stage (DS), location in the host (L), life cycle type (LC), and known definitive host/s (DH).

**Parasites**	***N***	**DS**	**L**	**LC**	**DH**
**Copepoda**					
*Cecrops latreillii* Leach, 1816	12	Adult	Gills	Direct	Molidae fishes
*Lepeophtheirus nordmanni* (Milne Edwards, 1836)	7	Adult	Skin	Direct	Molidae fishes
**Monogenea**					
*Capsala martinieri* Bosc, 1811	2	Adult	Skin	Direct	Molidae fishes
**Digenea**					
*Accacladocoelium macrocotyle* (Diesing, 1858) Robinson, 1934	2	Adult	Intestine	Indirect	Molidae fishes
*Accacladocoelium nigroflavum* (Rudolphi, 1819) Robinson, 1934	1	Adult	Intestine	Indirect	*Mola* spp.
*Accacoelium contortum* (Rudolphi, 1819) Looss, 1899	6	Adult	Gills, rectum	Indirect	Molidae fishes
**Cestoda**					
*Anchistrocephalus microcephalus* (Rudolphi, 1819)	5	Adult	Intestine	Indirect	Molidae fishes
*Hepatoxylon trichiuri* (Holten, 1802) Bosc, 1811	1	Plerocercoid larva	Body cavity	Indirect	Pelagic sharks
*Gymnorhynchus isuri* Robinson, 1959	9	Plerocercoid larva	Liver	Indirect	Pelagic sharks
**Nematoda**					
*Anisakis simplex* (s.s.) (Rudolphi, 1809)	1	Larva type I	Intestinal serosa	Indirect	Cetaceans

### Molecular Analysis for Selected Larval Parasites

According to the obtained sequences at the complete small subunit (ssrDNA) and the partial large subunit (1srDNA) ribosomal RNA gene, a larva was identified as *G. isuri*. The lsrDNA sequences (1,300 bp) and the ssrDNA sequences (1,968 bp) matched at 99–100% with the sequences of *G. isuri* from GenBank (25).

**Figure 2:**
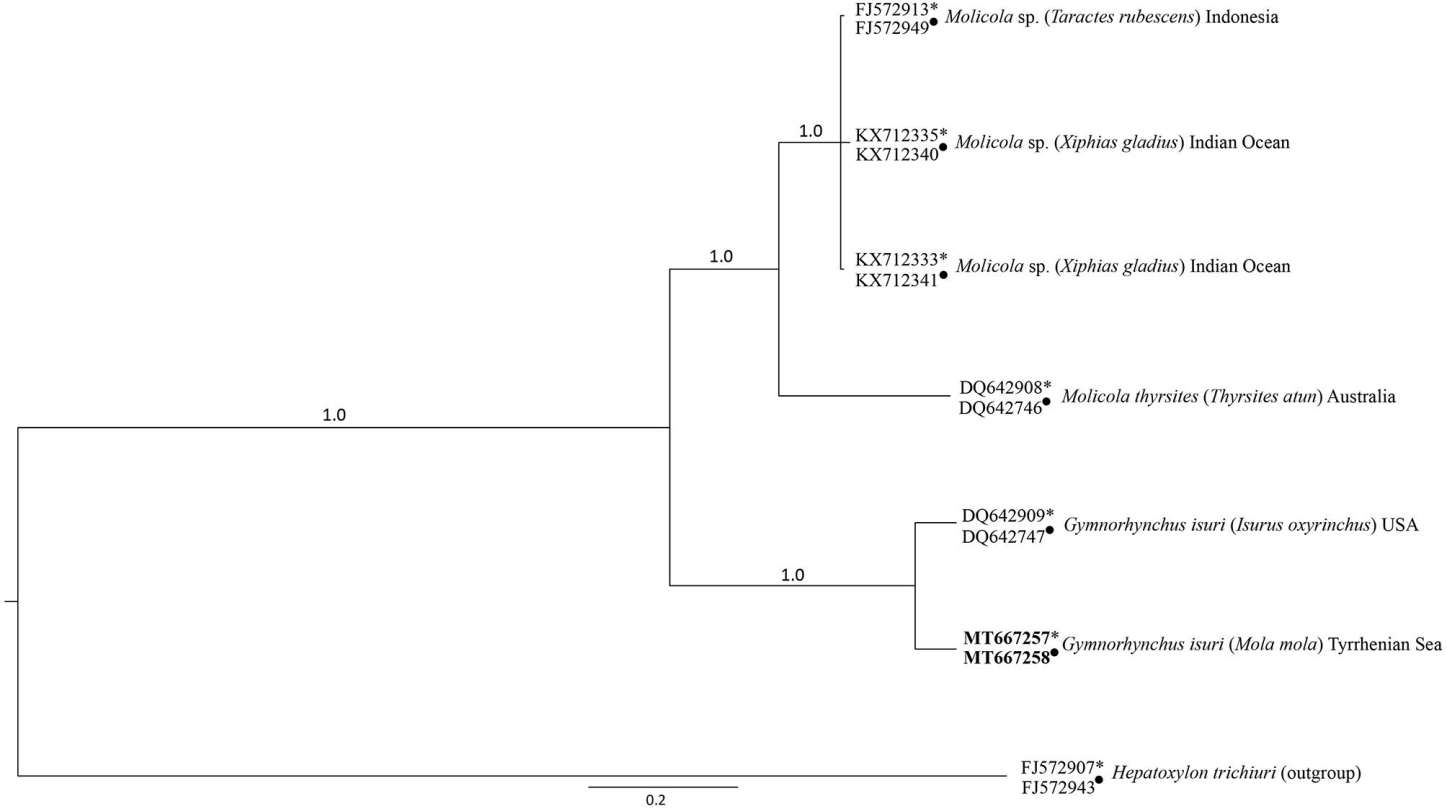
Phylogenetic condensed tree from Bayesian analyses based on 1srDNA and ssrDNA sequences obtained in the present study, in comparison with those available in GenBank at the same genes loci (1srDNA indicated with ∙, ssrDNA indicated with *) from cestodes of the Gymnorhynchidae Family. *Hepatoxylon trichiuri* was used as outgroup.

The BI obtained from the combined lsrDNA and the ssrDNA sequences dataset showed that the larva of *G. isuri* clustered together with the reference sequence of *G. isuri* from GenBank (25) (Figure 2). The obtained lsrDNA and ssrDNA sequences were deposited in GenBank under the accession numbers MT667258 and MT667257, respectively.

According to the sequences of 629 bp in length of the mtDNA *cox2* gene locus, the *Anisakis* type I larva was assigned to the species *A. simplex* (s.s.). The obtained sequence matched 100% with the *A. simplex* (s.s.) sequences from GenBank. The obtained *cox2* sequence was deposited in GenBank under the accession number MT667259.

## Discussion

*Mola mola* is usually considered a highly migratory fish, characterized by daily vertical movements following zooplankton preys and generally jellyfishes (1). Its horizontal movements are linked to seasonal variations of sea surface temperature and forage availability, with migrations shown to exceed 1,000 km in the northeast and northwest Atlantic (1, 35, 36). Recent studies suggest an ontogenetic shift in diet with age, with smaller sunfishes feeding on benthic invertebrates and larger individuals feeding on gelatinous zooplankton occurring in the water column (37–39). The present finding agrees with these statements, as our juvenile sunfish specimen (43 cm total length, corresponding to ca. 15-months of age according to captive growth rates for *M. mola*) [see (40)] was collected with a fishing line near the seabed, thus suggesting that the sunfish was foraging in the benthic zone.

The ecto-parasites found in the specimen from off Ischia Island were considered all *Mola* specialists with a direct life cycle, and have been found through the whole geographical range of their hosts, while the endo-parasites were all trophically transmitted with a heteroxenous life cycle (6, 24–27, 41, 42). All parasites, except *G. isuri* and *A. simplex* (s.s.), were previously found in *Mola* species from the Mediterranean Sea (6, 41, 42).

In the present study, we have identified two parasite species [*G. isuri* and *A. simplex* (s.s.)] that potentially could be used as biological tags, having both some features able to producing data for fish stock identification and for a better understanding of the host migration routes [see (9–11)].

Nematodes of the genus *Anisakis* infect a wide range of marine hosts (with cetaceans as definitive hosts) including at least 12 species of dolphins, porpoises and whales (43). Small crustaceans (Euphausiidae), fishes, squids, and other invertebrates serve as first and second intermediate or paratenic hosts (43, 44). *Anisakis* larvae have been previously reported in *Mola* species from Spain, New Zealand, and Chile (5, 6, 42), although no molecular analyses were performed to identify those larvae to species level. *Anisakis simplex* (s.s.) is known from the Atlantic and Pacific Oceans, and has its southern limit of distribution in the North-Eastern Atlantic waters along the Spanish-Portuguese Atlantic coast, being never recorded in the Mediterranean Sea except from the Alboran Sea, a transitional zone between Atlantic and Mediterranean [see (43)], and in pelagic fishes like the Atlantic mackerel *Scomber scombrus* Linnaeus, 1758 and the Atlantic bluefin tuna *Thunnus thynnus* Linnaeus, 1758 in the Eastern Mediterranean (45, 46); however, it has been suggested that the latter findings were related to the large migratory routes of those intermediate/paratenic fish hosts (45, 46).

Trypanorhynchan cestodes use copepods as first and other invertebrates or small fishes as second intermediate hosts (47, 48). Species of the genera *Gymnorhynchus* occur as adults in pelagic sharks, with the larval stages infecting a wide range of teleosts and sharks (47, 48). The genus *Gymnorhynchus* includes two species, namely *G. gigas* and *G. isuri* (48). To the best of our knowledge, only *G. gigas* has to date been reported from the Mediterranean basin in the swordfish *Xiphias gladius* Linnaeus, 1758, the Ray's bream *Brama brama* (Bonnaterre, 1788), and the silver scabbardfish *Lepidopus caudatus* (Euphrasen, 1788) (49, 50). *Gymnorhynchus isuri* is known from the north, southwestern, and northeast Atlantic and the Tasmanian Sea in the southwestern Pacific, infecting the shortfin mako *Isurus oxyrinchus* Rafinesque, 1810 and the blue shark *Prionace glauca* (Linnaeus, 1758) (30, 51, 52), and no record exists of *G. isuri* in *Mola* species anywhere.

A possible scenario seems to suggest that the sunfish specimen here analyzed had migrated from Atlantic Ocean waters into the Mediterranean Sea or at least from Alboran Sea waters. This hypothesis is based on the finding of parasite species typically known from Atlantic waters. No data exist on migration in juvenile *M. mola*, nor on Molidae in general within the Mediterranean Sea or from the Atlantic Ocean to the Mediterranean and *vice versa*, except for a study showing movements from the Atlantic Iberian coast to the Alboran Sea (36). Published information on the parasite fauna of the target fish species highlighted assemblages characterized by species with wide geographic range. This suggests the hypothesis that host migration route may be responsible for the parasite range expansion, as suggested for other marine vertebrates, that migrate from the Atlantic to the Mediterranean sea waters and *vice versa* (11, 45, 46, 53).

Among trypanorhynchans, *Molicola horridus* (Goodsir, 1841) Dollfus, 1935 (Gymnorhynchidae) is often reported infecting the liver of *Mola* species (6, 41, 42). *Molicola* is closely related to *Gymnorhynchus* having a similar scolex morphology, surface ultrastructure, and tentacular armature, and members of both genera also cause similar pathological changes; however, whereas *M. horridus* is considered a specific parasite of *Mola* species, *G. isuri* is known from bramid fishes (48). As the plerocerci of these two genera are difficult to be differentiated by morphology only (encysted forms are often obtained damaged, dead and with tentacles retracted), there is the possibility that the two species were often mixed up or misidentified in the past literature. In fact, the most important diagnostic feature between the two genera is the arrangement of hooks on the external surface of tentacular armature, that is impossible to study in encysted dead larval stages (48). Indeed, in recent years, molecular tools for recognition of members of Gymnorhynchidae was successfully applied (30).

Regarding to the other helminths identified in the present study, the cestodes *H. trichiuri* (Sphyriocephalidae) and *A. microcephalus* (Triaenophoridae) parasitize as adults the intestine of pelagic sharks and Molidae fishes respectively (5, 6, 26, 41, 42, 47, 48). It is believed that copepods can serve as intermediate hosts for both cestode species (48, 54). Digeneans of the genera *Accacoelium*, and *Accacladocoelium* (Accacoeliidae) are specific parasites of Molidae fishes, that acquire the infection when feeding on nektonic organisms, and especially on cnidarians and ctenophores infected by accacoeliid metacercariae (24, 55).

In conclusion, traditional parasitological studies implemented by molecular tools are still needed to understand if host specificity exists for most of the parasites infecting *Mola* species. However, despite we have here analyzed only a single *M. mola* specimen, we have showed that this sunfish species serves as an intermediate host for two cestodes (*H. trichiuri* and *G. isuri*) and as a definitive host for the remaining parasites except *A. simplex* (s.s.), considered here as an accidental finding. In fact, due to low prevalence and intensity of infection or absence of this nematode larvae, as also reported in other studies from Mediterranean (6, 41, 42), it is reasonable to conclude that this fish ecologically likely represents a dead end for the parasite (56). For the reasons listed above, the potential use of *A. simplex* (s.s.) as biological tag for sunfishes remains doubtful. In contrast, the cestode *G. isuri* has a useful potential to be used as biological indicator and could be used to study the sunfish movements at least in the Mediterranean and adjacent areas. However, despite the present study reveals for the first time the occurrence of *G. isuri* in the Mediterranean Sea and indicates *M. mola* as a new intermediate host record for this cestode species, a molecular screening of specimen morphologically identified in the past literature and in museum collections as *M. horridus* could confirm if the present finding of *G. isuri* in the Mediterranean is a new or casual event or the species is already widespread, but undetected and/or misidentified, in the basin.

## Data Availability Statement

The datasets presented in this study can be found in online repositories. The names of the repository/repositories and accession number(s) can be found in the article/Supplementary Material.

## Ethics Statement

Ethical review and approval was not required for the animal study because the sunfish was collected during amateur fishing with no additional experimental catches being performed. According to Italian law DL16/92 and European directive 2010/63/EU, this study did not require a specific permit. Procedures for this study were performed in accordance with the guide for the care and use of animals by the Italian Ministry of Health.

## Author Contributions

MS: performed fish necropsy, collection, morphological identification of parasites, and wrote the paper. SM and MP: performed molecular and phylogenetic analyses of selected larval stages of parasites. DO and FC: performed molecular and phylogenetic analyses of the sunfish. All authors contributed to manuscript revision, read, and approved the submitted version.

## Conflict of Interest

The authors declare that the research was conducted in the absence of any commercial or financial relationships that could be construed as a potential conflict of interest.

## Supplementary Material

Supplementary Figure 1 Phylogenetic relationships in the genus *Mola* based on mtDNA *cox1* partial sequences downloaded from GenBank and BOLD (ID codes as in the Supplementary Table 1), and the specimen from off Ischia Island marked in bold. Numbers above/below branches represent bootstrap values (ML) and posterior probabilities values (BI). Scale bar represents nucleotide substitution. Poor support values (<50%, 0.90) are not shown. ^**^The putative misidentification (see Supplementary Table 1) is based on an incorrect entry in GenBank [see (2), where the same specimen is labeled as *Mola* sp. A = *Mola alexandrini*].

Supplementary Table 1 GenBank and Barcode of Life Data System mtDNA cox1 partial sequences used in the molecular systematic analyses and associated specimen data (locality obtained from web databases or relevant paper/s). ^*^The specimen from Ischia Island (central Tyrrhenian Sea, Mediterranean) analysed in the present study. ^**^The putative misidentification is based on an incorrect entry in GenBank (where the same specimen is labelled *Mola* sp. A = *Mola alexandrini*).

## References

[1] Pope E Hays G Thys T Doyle T Sims D Queiroz N The biology and ecology of the ocean sunfish *Mola mola*: a review of current knowledge and future research perspectives. Rev Fish Biol Fish. (2010) 20:471–87. 10.1007/s11160-009-9155-9

[2] Nyegaard M Sawai E Gemmell N Gillum J Loneragan NR Yamanoue Y Hiding in broad daylight: molecular and morphological data reveal a new ocean sunfish species (Tetraodontiformes: Molidae) that has eluded recognition. Zool J Linnean Soc. (2018) 182:631–58. 10.1093/zoolinnean/zlx040

[3] Sawai E Yamanoue Y Nyegaard M Sakai Y Redescription of the bump-head sunfish *Mola alexandrini* (Ranzani 1839), senior synonym of *Mola ramsayi* (Giglioli 1883), with designation of a neotype for *Mola mola* (Linnaeus 1758) (Tetraodontiformes: Molidae). Ichthyol Res. (2018) 65:142–60. 10.1007/s10228-017-0603-6

[4] Mangel JC Pajuelo M Pasara-Polack A Vela G Segura-Cobeña E Alfaro-Shigueto J The effect of Peruvian small-scale fisheries on sunfishes (Molidae). J Fish Biol. (2019) 94:77–85. 10.1111/jfb.13862 30421420

[5] de Figueiredo NC de Lima JTAX Freitas CI da Silva CG Checklist dos parasitos do peixe Lua (*Mola mola*: Molidae) no mundo. Pubvet. (2018) 12:1–9. 10.22256/pubvet.v12n3a42.1-9

[6] Ahuir-Baraja AE Yamanoue Y Kubicek L. First confirmed record of *Mola* sp. A in the western Mediterranean Sea: morphological, molecular and parasitological findings. J Fish Biol. (2017) 90:1133–41. 10.1111/jfb.13247 28105658

[7] Poulin R. Determinants of host-specificity in parasites of freshwater fishes. Int J Parasitol. (1992) 22:753–8. 10.1016/0020-7519(92)90124-4 1428509

[8] Poulin R Krasnov BR Mouillot D. Host specificity in phylogenetic and geographic space. Trends Parasitol. (2011) 27:355–61. 10.1016/j.pt.2011.05.003 21680245

[9] MacKenzie K Abaunza P Parasites as biological tags for stock discrimination of marine fish: a guide to procedures and methods. Fish Res. (1998) 38:45–56. 10.1016/S0165-7836(98)00116-7

[10] Catalano SR Whittington ID Donnellan SC Gillanders BM. Parasites as biological tags to assess host population structure: guidelines, recent genetic advances and comments on a holistic approach. Int J Parasitol Parasites Wildl. (2013) 3:220–6. 10.1016/j.ijppaw.2013.11.001 25197624 PMC4152261

[11] Mattiucci S Cimmaruta R Cipriani P Abaunza P Bellisario B Nascetti G. Integrating *Anisakis* spp. parasites data and host genetic structure in the frame of a holistic approach for stock identification of selected Mediterranean Sea fish species. Parasitology. (2015) 142:90–108. 10.1017/S0031182014001103 25145788

[12] Santoro M Di Nocera F Iaccarino D Cipriani P Guadano Procesi I Maffucci F. Helminth parasites of the dwarf sperm whale *Kogia sima* (Cetacea: Kogiidae) from the Mediterranean Sea, with implications on host ecology. Dis Aquat Organ. (2018) 129:175-82. 10.3354/dao03251 30154277

[13] Osca D Tanduo V Tiralongo F Giovos I Almabruk SAA Crocetta F The indo-pacific sergeant *abudefduf vaigiensis* (Quoy & Gaimard, 1825) (Perciformes: Pomacentridae) in libya, South-central mediterranean sea. J Mar Sci Eng. (2020) 8:14 10.3390/jmse8010014

[14] Morgulis A Coulouris G Raytselis Y Madden TL Agarwala R Schäffer AA. Database indexing for production MegaBLAST searches. Bioinformatics. (2008) 24:1757–64. 10.1093/bioinformatics/btn322 18567917 PMC2696921

[15] Abascal F Zardoya R Telford MJ. TranslatorX: multiple alignment of nucleotide sequences guided by amino acid translations. Nucleic Acids Res. (2010) 38(Suppl.2):W7–13. 10.1093/nar/gkq291 20435676 PMC2896173

[16] Katoh K Standley DM. MAFFT multiple sequence alignment software version 7: improvements in performance and usability. Mol Biol Evol. (2013) 30:772–80. 10.1093/molbev/mst010 23329690 PMC3603318

[17] Glez-Peña D Gómez-Blanco D Reboiro-Jato M Fdez-Riverola F Posada D. ALTER: program-oriented conversion of DNA and protein alignments. Nucleic Acids Res. (2010) 38:14–8. 10.1093/nar/gkq321 20439312 PMC2896128

[18] Akaike H Information theory and an extension of the maximum likelihood principle. In: Petrov BN, Csaki F, editors. Second International Symposium on Information Theory. Budapest: Academiai Kiado (1973). p. 267–81.

[19] Lanfear R Calcott B Ho SYW Guindon S. PartitionFinder: combined selection of partitioning schemes and substitution models for phylogenetic analyses. Mol Biol Evol. (2012) 29:1695–701. 10.1093/molbev/mss020 22319168

[20] Stamatakis A RAxML version 8: a tool for phylogenetic analysis and post-analysis of large phylogenies. Bioinformatics. (2014) 30:1312–3. 10.1093/bioinformatics/btu033 24451623 PMC3998144

[21] Ronquist F Huelsenbeck JP. MrBayes 3: Bayesian phylogenetic inference under mixed models. Bioinformatics. (2003) 19:1572–4. 10.1093/bioinformatics/btg180 12912839

[22] Felsenstein J. Confidence limits on phylogenies: an approach using the bootstrap. Evolution. (1985) 39:783–91. 10.1111/j.1558-5646.1985.tb00420.x 28561359

[23] Santoro M Mattiucci S Cipriani P Bellisario B Romanelli F Cimmaruta R Parasite communities of icefish (*Chionodraco hamatus*) in the Ross Sea (Antarctica): influence of the host sex on the helminth infracommunity structure. PLoS ONE. (2014) 9:e88876 10.1371/journal.pone.0088876 24558440 PMC3928312

[24] Bray RA Gibson DI The accacoeliidae (Digenea) of fishes from the North-East Atlantic. Bull Brit Mus Nat Hist Zool. (1977) 31:53–99.

[25] Kabata Z Copepoda and branchiura. In: Margolis L, Kabata Z, editors. Guide to the parasites of fishes of Canada. Part II – Crustacea: Can Spec Publ Fish Aquat Sci. (1988). p. 3–127.

[26] Khalil LF Jones A Bray RA. Keys to the Cestode Parasites of Vertebrates. Wallingford: CAB International. (1994). p. 768.

[27] Lamothe-Argumedo R Nuevo arreglo taxonómico de la subfamilia Capsalinae (Monogenea: Capsalinae), clave para los géneros y dos combinaciones nuevas. An Inst Biol Univ Nac Autón Mexico Ser Zool. (1997) 68:207–23.

[28] Littlewood DTJ Cribb TH Olson PD Bray RA Platyhelminth phylogenetics-a key to understanding parasitism? Belg J Zool. (2001) 131:35–46.

[29] van der Auwera G Chapelle S De Wachter R. Structure of the large ribosomal subunit RNA of *Phytophthora megasperma*, and phylogeny of the oomycetes. FEBS Lett. (1994) 338:133–6. 10.1016/0014-5793(94)80350-1 8307170

[30] Palm HW Waeschenbach A Olson PD Littlewood DT Molecular phylogeny and evolution of the trypanorhyncha Diesing, 1863 (Platyhelminthes: Cestoda). Mol Phylogenet Evol. (2009) 52:351–67. 10.1016/j.ympev.2009.01.019 19489123

[31] Larkin MA Blackshields G Brown NP Chenna R McGettigan PA McWilliam H. Clustal W and clustal X version 2.0. Bioinformatics. (2007) 23:2947–8. 10.1093/bioinformatics/btm404 17846036

[32] Vaidya G Lohman DJ Meier R SequenceMatrix: concatenation software for the fast assembly of multi-gene datasets with character set and codon information. Cladistics. (2011) 27:171–80. 10.1111/j.1096-0031.2010.00329.x 34875773

[33] Posada D Buckley TR Model selection and model averaging in phylogenetics: advantages of Akaike information criterion and Bayesian approaches over likelihood ratio tests. Syst Biol. (2004) 53:793–808. 10.1080/10635150490522304 15545256

[34] Mattiucci S Cipriani P Webb SC Paoletti M Marcer F Bellisario B Genetic and morphological approaches distinguish the three sibling species of the *Anisakis simplex* species complex, with a species designation as *Anisakis berlandi* n. sp. for A. simplex sp. C (Nematoda: Anisakidae). J Parasitol. (2014) 100:199–214. 10.1645/12-120.1 24224764

[35] Potter IF Galuardi B Howell WH Horizontal movement of ocean sunfish, *Mola mola*, in the northwest Atlantic. Mar Biol. (2011) 158:531–40. 10.1007/s00227-010-1578-2

[36] Sousa L Queiroz N Mucientes G Humphries NE Sims DW Environmental influence on the seasonal movements of satellite-tracked ocean sunfish *Mola mola* in the north-east Atlantic. Anim Biotelemetry. (2016) 4:7 10.1186/s40317-016-0099-2

[37] Syväranta J Harrod C Kubicek L Cappanera V Houghton JD Stable isotopes challenge the perception of ocean sunfish *Mola mola* as obligate jellyfish predators. J Fish Biol. (2012) 80:225–31. 10.1111/j.1095-8649.2011.03163.x 22220901

[38] Sousa L Xavier R Costa V Humphries NE Trueman C. DNA barcoding identifies a cosmopolitan diet in the ocean sunfish. Sci Rep. (2016) 6:28762. 10.1038/srep28762 27373803 PMC4931451

[39] Nakamura I Sato K Ontogenetic shift in foraging habit of ocean sunfish *Mola mola* from dietary and behavioral studies. Mar Biol. (2014) 161:1263–73. 10.1007/s00227-014-2416-8

[40] Nakatsubo T Hirose H Growth of captive ocean sunfish, *Mola mola*. Suisan Zoshoku. (2007) 55:403–7.

[41] Gustinelli A Nardini G Aureli G Trentini M Affronte M Fioravanti ML Parasitofauna of *Mola mola* (Linnaeus, 1758) from Italian seas. Biol Mar Med. (2006) 13:872–6.

[42] Ahuir-Baraja AE Estudio parasitológico del pez luna, Mola mola (l.), en el mediterráneo occidental. (PhD Thesis), Universitat de València (2012).

[43] Mattiucci S Cipriani P Levsen A Paoletti M Nascetti G Molecular epidemiology of *Anisakis* and Anisakiasis: an ecological and evolutionary road map. Adv Parasitol. (2018) 99:93–263. 10.1016/bs.apar.2017.12.001 29530312

[44] Klimpel S Palm HW Rückert S Piatkowski U. The life cycle of *Anisakis simplex* in the norwegian deep (northern North Sea). Parasitol Res. (2004) 94:1–9. 10.1007/s00436-004-1154-0 15278439

[45] Levsen A Cipriani P Mattiucci S Gay M Hastie LC MacKenzie K *Anisakis* species composition and infection characteristics in Atlantic mackerel, *Scomber scombrus*, from major European fishing grounds - reflecting changing fish host distribution and migration pattern. Fish Res. (2018) 202:112–21. 10.1016/j.fishres.2017.07.030

[46] Mladineo I Trumbić Z Radonić I Vrbatović A Hrabar J Bušelić I *Anisakis simplex* complex: ecological significance of recombinant genotypes in an allopatric area of the Adriatic Sea inferred by genome-derived simple sequence repeats. Int J Parasitol. (2017) 47:215–23. 10.1016/j.ijpara.2016.11.003 28057461

[47] Campbell RA Beveridge I Order trypanorhyncha Diesing, 1863. In: Khalil LF, Jones A, Bray RA, editors. Keys to the Cestode Parasites of Vertebrates. Wallingford: CAB International (1994). p. 51–148.

[48] Palm HW The Trypanorhyncha Diesing, 1863. Bogor: IPB-PKSPL Press (2004). p. 710.

[49] Manfredi MT Gandini G Traldi G Infestazione muscolare da larve di cestodi Trypanorhyncha in pesce spada (*Xiphias gladius*). Atti Soc Ital Sci Vet. (1993) 47:765–7.

[50] Giarratana F Muscolino D Beninati C Ziino G Giuffrida A Trapani M *Gymnorhynchus gigas* in *Lepidopus caudatus* (Actinopterygii: perciformes: trichiuridae): prevalence and related effects on fish quality. Czech J Food Sci. (2014) 32:320–5. 10.17221/330/2013-CJFS

[51] Knoff M São Clemente SC Pinto RM Lanfredi RM Gomes DC Redescription of *Gymnorhynchus isuri* (Cestoda: Trypanorhyncha) from *Isurus oxyrinchus* (Elasmobranchii: Lamnidae). Folia Parasitol. (2007) 54:208–14. 10.14411/fp.2007.028 19245192

[52] Penadés-Suay J Tomás J Merchán M Aznar FJ. Intestinal helminth fauna of the shortfin mako *Isurus oxyrinchus* (Elasmobranchii: Lamnidae) in the northeast Atlantic Ocean. Dis Aquat Organ. (2017) 123:45–54. 10.3354/dao03081 28177292

[53] Santoro M Di Nocera F Iaccarino D Lawton SP Cerrone A Degli Uberti B. Pathology and molecular analysis of *Hapalotrema mistroides* (Digenea: Spirorchiidae) infecting a Mediterranean loggerhead turtle *Caretta caretta*. Dis Aquat Organ. (2017) 124:101–8. 10.3354/dao03117 28425423

[54] Kuchta R Scholz T Bray RA. Revision of the order Bothriocephalidea Kuchta, Scholz, Brabec & Bray, 2008 (Eucestoda) with amended generic diagnoses and keys to families and genera. Syst Parasitol. (2008) 71:81–136. 10.1007/s11230-008-9153-7 18716900

[55] Gibson DI Family accacoeliidae Odhner, 1911. In: Gibson DI, Jones A, Bray RA, editors. Keys to the Trematoda Volume 1. London, UK: CABI Publishing and The Natural History Museum (2002). p. 341–7. 10.1079/9780851995472.0341

[56] Santoro M Mattiucci S Paoletti M Liotta A Uberti BD Galiero G Molecular identification and pathology of *Anisakis pegreffii* (Nematoda: Anisakidae) infection in the Mediterranean loggerhead sea turtle (*Caretta caretta*). Vet Parasitol. (2010) 174:65–71. 10.1016/j.vetpar.2010.08.018 20850929

