# Supplementary figures and images for: New Parasite Records for the Sunfish Mola mola in the Mediterranean Sea and Their Potential Use as Biological Tags for Long-Distance Host Migration
Source: Front Vet Sci. 2020 Oct 19;7:579728. doi: 10.3389/fvets.2020.579728 (PMC7641614; doi:10.3389/fvets.2020.579728)

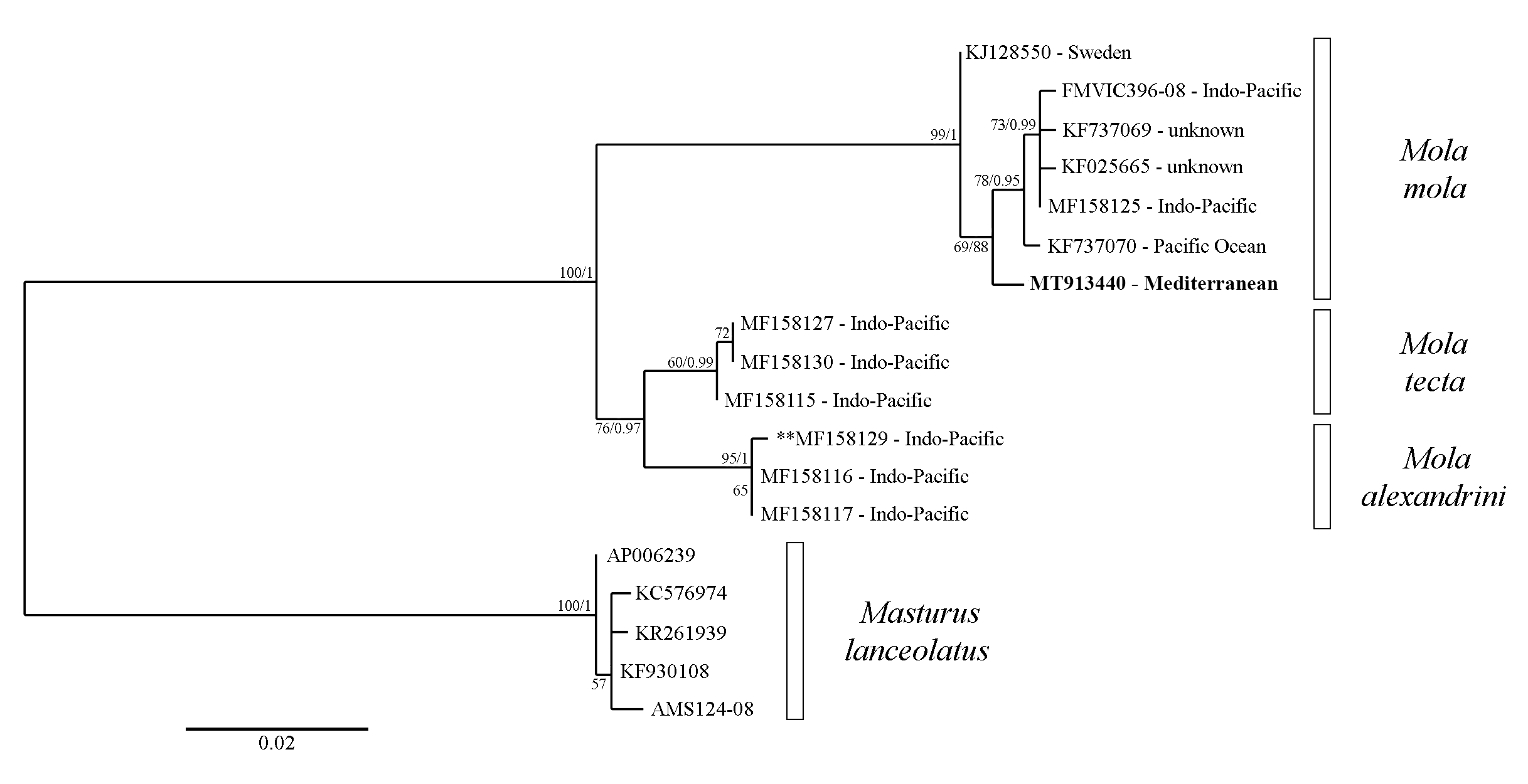

Supplement: Supplementary Figure 1 — Phylogenetic relationships in the genus Mola based on mtDNA cox1 partial sequences downloaded from GenBank and BOLD (ID codes as in the Supplementary Table 1), and the specimen from off Ischia Island marked in bold. Numbers above/below branches represent bootstrap values (ML) and posterior probabilities values (BI). Scale bar represents nucleotide substitution. Poor support values (<50%, 0.90) are not shown. **The putative misidentification (see Supplementary Table 1) is based on an incorrect entry in GenBank [see (2), where the same specimen is labeled as Mola sp. A = Mola alexandrini]. [file Image_1.TIF]
