# Supplementary material for: New Parasite Records for the Sunfish Mola mola in the Mediterranean Sea and Their Potential Use as Biological Tags for Long-Distance Host Migration
Source: Front Vet Sci. 2020 Oct 19;7:579728. doi: 10.3389/fvets.2020.579728 (PMC7641614; doi:10.3389/fvets.2020.579728)
Supplement: Supplementary Table 1 — GenBank and Barcode of Life Data System mtDNA cox1 partial sequences used in the molecular systematic analyses and associated specimen data (locality obtained from web databases or relevant paper/s). *The specimen from Ischia Island (central Tyrrhenian Sea, Mediterranean) analysed in the present study. **The putative misidentification is based on an incorrect entry in GenBank (where the same specimen is labelled Mola sp. A = Mola alexandrini). [file Table_1.docx]

**SUPPLEMENTARY TABLE 1** GenBank and Barcode of Life Data System cox1 partial sequences used in the molecular systematic analyses and associated specimen data (locality obtained from web databases or relevant paper/s). *The specimen from Ischia Island (central Tyrrhenian Sea, Mediterranean) analysed in the present study. **The putative misidentification is based on an incorrect entry in GenBank (see Nyegaard et al. 2018: Table S1, where the same specimen is labelled *Mola* sp. A = *Mola alexandrini*).

| **Species identification** | | | **ID code** | | **Locality** | **Reference** |
| --- | --- | --- | --- | --- | --- | --- |
| **This study** | **GenBank** | **Bold** | **GenBank** | **Bold** |  |  |
| *Mola mola** |  |  | MT913440 |  | Mediterranean: Ischia Island | Present paper |
| *Mola mola* | *Mola mola* |  | KJ128550 |  | Sweden | GenBank |
| *Mola mola* | *Mola mola* |  | KF737069 |  | - | GenBank |
| *Mola mola* | *Mola mola* |  | KF025665 |  | - | Santini et al. 2013 |
| *Mola mola* | *Mola* sp. B |  | MF158125 |  | Indo-Pacific: New Zealand | Nyegaard et al. 2018 |
| *Mola mola* | *Mola mola* |  | KF737070 |  | Pacific Ocean: California | GenBank |
| *Mola mola* |  | *Mola mola* |  | FMVIC396-08 | Indo-Pacific: Australia | Nyegaard et al. 2018 |
| *Mola tecta* | *Mola tecta* |  | MF158127 |  | Indo-Pacific: New Zealand | Nyegaard et al. 2018 |
| *Mola tecta* | *Mola tecta* |  | MF158130 |  | Indo-Pacific: New Zealand | Nyegaard et al. 2018 |
| *Mola tecta* | *Mola tecta* |  | MF158115 |  | Indo-Pacific: New Zealand | Nyegaard et al. 2018 |
| *Mola alexandrini* | *Mola tecta*** |  | MF158129 |  | Indo-Pacific: New Zealand | Nyegaard et al. 2018 |
| *Mola alexandrini* | *Mola* sp. A |  | MF158116 |  | Indo-Pacific: New Zealand | Nyegaard et al. 2018 |
| *Mola alexandrini* | *Mola* sp. A |  | MF158117 |  | Indo-Pacific: New Zealand | Nyegaard et al. 2018 |
| *Masturus lanceolatus* | *Masturus lanceolatus* |  | AP006239 |  | - | Yamanoue et al. 2004 |
| *Masturus lanceolatus* | *Masturus lanceolatus* |  | KC576974 |  | India | Prakash et al. 2016 |
| *Masturus lanceolatus* | *Masturus lanceolatus* |  | KR261939 |  | Vietnam | GenBank |
| *Masturus lanceolatus* | *Masturus lanceolatus* |  | KF930108 |  | USA: Florida | GenBank |
| *Masturus lanceolatus* |  | *Masturus lanceolatus* |  | AMS124-08 | - | Bold |
